# Altered Expression of DAAM1 and PREP Induced by Cadmium Toxicity Is Counteracted by Melatonin in the Rat Testis

**DOI:** 10.3390/genes12071016

**Published:** 2021-06-30

**Authors:** Massimo Venditti, Mariem Ben Rhouma, Maria Zelinda Romano, Imed Messaoudi, Russel J. Reiter, Sergio Minucci

**Affiliations:** 1 Dipartimento di Medicina Sperimentale, Sez. Fisiologia Umana e Funzioni Biologiche Integrate “F. Bottazzi”, Università degli Studi della Campania “Luigi Vanvitelli”, via Costantinopoli, 16, 80138 Napoli, Italy; massimo.venditti@unicampania.it (M.V.); mariazelinda.romano@unicampania.it (M.Z.R.); 2 Laboratoire LR11ES41 Génétique Biodiversité et Valorisation des Bio-Ressourcés, Institut Supérieur de Biotechnologie de Monastir, Université de Monastir, Rue Taher Haddad, Monastir 5000, Tunisia; benrhoumamariem98@gmail.com (M.B.R.); imed_messaoudi@yahoo.fr (I.M.); 3 Department of Cellular and Structural Biology, UT Health Science Center, 7703 Floyd Curl Drive, San Antonio, TX 78229, USA; reiter@uthscsa.edu

**Keywords:** melatonin, cadmium, testis, oxidative stress, endocrine disrupters, cytoskeleton, DAAM1, PREP

## Abstract

Cadmium (Cd) is one of the most toxic pollutants for health due to its accumulation in several tissues, including testis. This report confirms that Cd increased oxidative stress and apoptosis of germ and somatic cells and provoked testicular injury, as documented by biomolecular and histological alterations, i.e., CAT and SOD activity, the protein level of steroidogenic enzymes (StAR and 3β-HSD), and morphometric parameters. Additionally, it further documents the melatonin (MLT) coadministration produces affects in mitigating Cd-induced toxicity on adult rat testis, as demonstrated by the reduction of oxidative stress and apoptosis, with reversal of the observed histological changes; moreover, a role of MLT in partially restoring steroidogenic enzymes expression was evidenced. Importantly, the cytoarchitecture of testicular cells was perturbed by Cd exposure, as highlighted by impairment of the expression and localization of two cytoskeleton-associated proteins DAAM1 and PREP, which are involved in the germ cells’ differentiation into spermatozoa, altering the normal spermatogenesis. Here, for the first time, we found that the co-treatment with MLT attenuated the Cd-induced toxicity on the testicular DAAM1 and PREP expression. The combined findings provide additional clues about a protective effect of MLT against Cd-induced testicular toxicity by acting on DAAM1 and PREP expression, encouraging further studies to prove its effectiveness in human health.

## 1. Introduction

Reproduction is an essential biological feature that increases the number of living beings and is necessary for the survival and the evolutionary continuity of the species. For this, the production and differentiation of good quality gametes is a fundamental for reproductive success. However, it is well known that about 15% of couples worldwide are affected with infertility, and approximately half of the cases can be attributed to male sterility [1]. Despite notorious genetic and physio-pathological causes provoke male infertility, recent research are demonstrating that environmental factors play a prominent role in the determination of this condition [2,3,4]. In fact, in the more industrialized countries, there is a massive release in all the compartments of environments of many kinds of pollutants that may affect human health, inducing spermatogenic and hormonal alterations and acting as endocrine disruptor chemicals [5], leading to infertility [6]. 

Research focuses particularly on the effects of heavy metals, but cadmium (Cd) is pre-eminently studied because of its widespread prevalence in the general population, above all via food and cigarette smoke, and for its high testicular toxicity [7,8]. In fact, many reports demonstrated that Cd affects animal and human spermatogenesis, acting at different levels: disturbing the hypothalamus-pituitary-testis (HPT) axis; altering seminiferous tubules, blood-testis barrier, and testicular endothelium; and finally resulting in reduced spermatozoa (SPZ) quantity and quality [7,8]. All the above-mentioned effects of Cd on the testis have been related, at the cellular and molecular level, to an increase of oxidative stress, augmented tissue apoptosis and necrosis, and alteration of steroidogenesis and spermatogenesis; all the combined effects result in the reduction of normal SPZ morphology and motility and, finally, lead to reproductive disorders [9,10,11]. 

Spermatogenesis is a highly dynamic, proliferative, and differentiative process, which occurs thanks to the cytoskeleton elements of both germinal and somatic cells that contribute to produce mature gametes, which are able to fertilize [12,13]. 

In this scenario, it is remarkable the role played by the cytoskeleton-associated factors that ensure the correct organization and dynamics of cytoskeleton filaments fundamental for their activity [14].

In this regard, in our previous works, the involvement of two cytoskeleton-related proteins, dishevelled-associated activator of morphogenesis 1 (DAAM1) and prolyl endopeptidase (PREP), in spermatogenesis of rat testis was demonstrated [15,16,17,18]. Moreover, we evaluated the effects of maternal Cd exposure during gestation and lactation in male offspring on DAAM1 and PREP expression and localization in the adult rat testis as well as studying the role of zinc in counteracting the toxic effects that Cd induced [19,20]. 

Considering the remarkable effect of Cd on male fertility, many papers reported the importance of substances that may prevent, counteract, or ameliorate its toxicity to preserve a proper gamete production. Recently, among them, we focused our attention on zinc [19,20] and melatonin (MLT), evaluating their counteractive effects not only in the testis, but also in other tissues targeted by Cd toxicity, such as bone [21] and ovary [22].

MLT and indolamine are secreted by the pineal gland in circadian rhythm, which has been extensively proven to regulate the reproductive activity of seasonal breeding animals [23,24]. MLT effectiveness in reducing Cd (and other toxicants) -induced toxicity is mainly due to its antioxidant, such as free oxygen and nitrogen-based radicals’ scavenger, and antiapoptotic properties [25,26,27,28].

Thus, to expand upon the knowledge of the effects of Cd-induced toxicity, two different approaches compared to our previous works [19,20] were here employed: (1) male adult rats were orally treated with Cd to evaluate its direct effect(s) on mature gonads and on steroidogenesis; (2) the beneficial influence of another antioxidant molecule, MLT, was evaluated to augment the number of strategies that may be useful to counteract/prevent Cd reprotoxicity. Therefore, here, we used oral Cd/MLT administration to explore their consequences on the testicular physiopathology, evaluating several factors, including antioxidant enzyme activities, the apoptotic rate, and the protein level of steroidogenic enzymes, with a special attention to changes in the cytoskeletal-associated proteins DAAM1 and PREP.

## 2. Materials and Methods

### 2.1. Animals and Experimental Design

Twenty-four Wistar male rats, aged 2 months old and weighing 225 ± 36 g were kept in individual stainless-steel cages under controlled conditions of light (12:12 h light/dark), temperature (22 ± 2 °C), and humidity (55 ± 20%). Food and water were given *ad libitum*. Rats were randomly divided into four groups (n = 6 each): (1) control, (2) Cd-treated (50 mg CdCl_2_/L; Sigma-Aldrich), (3) MLT-treated (3 mg/L; Sigma-Aldrich); and (4) Cd + MLT-treated (50 mg CdCl_2_/L + 3 mg MLT/L). Cd and MLT were administered in the drinking water. The MLT stock solution was prepared in ethanol; final ethanol concentration in the drinking water was 0.015%. The 24 h consumption of drinking water and body weight were monitored during the whole experiment. Drinking water consumption and daily Cd and MLT intake were investigated according to the method described by Brzoska and Moniuszko-Jakoniuk [29]. Treatment with Cd and/or MLT did not influence the volume of water consumed by animals of the different groups (an average of 25 mL/day). Groups 1 and 2 received an equivalent amount of ethanol in drinking water. The used concentration of Cd and MLT were chosen according to literature [21,22,30]. To protect MLT from light, water bottles were changed twice weekly and covered with aluminum foil. Treatment lasted 40 days and, at the end of the period, animals were anesthetized with chloral hydrate and sacrificed. For each animal, testes were dissected, and right testis was immersed in 10% neutral buffered formalin for histological studies, while the left was kept at −80 °C for biomolecular studies. The experimental procedure was approved by the Ethics Committee for Research in life science and health of the Higher Institute of Biotechnology of Monastir (CER-SVS/ISBM- protocol 022/2020) and was carried out accordingly to the UNESCO Recommendation Concerning Science and Scientific Research (1974, 2017).

### 2.2. Evaluation of Testicular CAT and SOD Activities

Catalase (CAT) activity was assayed by the method of Claiborne [31], while superoxide dismutase (SOD) activity was measured by the method of Marklund and Marklund [32]. CAT and SOD activities were expressed as units per milligram of protein (U/mg of protein). 

### 2.3. Thiobarbituric Acid-Reactive Species (TBARS) Levels Assessment

Testicular TBARS levels were assayed according to Lama et al. [33]. Results were expressed as TBARS µM/µg of extracted protein. Each measurement was performed in triplicate.

### 2.4. Histology and TUNEL Assay

The fixed testes were dehydrated in increasing alcohol concentrations before paraffin embedding. Five-μm thick serial sections were stained with hematoxylin/eosin. For histopathological evaluation, 25 seminiferous tubules/animal, for a total of 150 tubules per group, were counted under light microscope (Leica DM 2500, Leica Microsystems, Wetzlar, Germany). Photographs were taken using the Leica DFC320 R2 digital Camera (Leica Microsystems, Wetzlar, Germany).

Apoptotic cells were investigated by the TUNEL-assay using DeadEnd™ Fluorometric TUNEL System (#G3250; Promega Corp., Madison, WI, USA) following manufacturer’s protocol. Sections were then counterstained with Vectashield+DAPI (#H-1200-10; Vector Laboratories, Peterborough, UK) to mark cell nuclei and with PNA lectin to mark the acrosome. The sections were observed and captured with the optical microscope (Leica DM 5000 B+CTR 5000, Leica Microsystems, Wetzlar, Germany) with UV lamp and saved with IM 1000 software.

### 2.5. Reverse Transcription Polymerase Chain Reaction (RT-PCR)

Total RNA was extracted from testicular samples using Trizol reagent (HiMedia Laboratories GmbH; Einhausen, Germany). The quantity (ng/mL) and purity (260/280 and 260/230 ratios) of total RNA were assessed using a NanoDrop 2000 spectrophotometer (Thermo, Waltham, MA, USA). First-strand cDNA was synthesized using 3 μg of total RNA in a total volume of 25 μL according to manufacturer’s instructions (#G592, abmgood; Richmond, BC, Canada). A total of 2 μL of the obtained cDNA template were then used for the PCR reaction [34]. The amplifications were carried out for 30 cycles, with denaturation at 94 °C for 30 s, annealing for 45 s, and extension at 72 °C for 45 s (for details, see Table S1). Amplification products, electrophoresed on 1.2% agarose gel, were quantified by densitometric analysis carried out using GELDOC1,00-UV system (Biorad, Hercules, CA, USA). The relative amount of the *Daam1* and *Prep* mRNAs was calculated by the *Daam1*/*Act* and *Prep*/*Act* ratio values and graphed as optical density (OD) units. Three independent assays were carried out to assess the statistical significance. 

### 2.6. Protein Extraction and Western Blotting (WB) Analysis

Proteins were extracted from the testis according to Ergoli et al. [35]. Forty micrograms of the protein extracts were separated by 9% SDS–PAGE and transferred to Hybond-P polyvinylidene difluoride membranes (#GE10600023; Amersham Pharmacia Biotech, Buckinghamshire, UK) at 280 mA for 2.5 h at 4 °C. For details concerning the used antibodies, see Table S1. The immunocomplexes were detected using the enhanced chemiluminescence (ECL)-WB detection system. ImageJ software (version 1.53g) was used to analyze all bands. WB was performed in triplicate.

### 2.7. Immunofluorescence (IF) Analysis

For IF staining, testis sections were processed according to Venditti et al. [36]. Permeabilization with PBS pH 7.4 containing 0.1% Triton-X-100 for 30 min was performed for the proteins not located on the plasma membrane. Later, sections were incubated with primary antibodies overnight at 4 °C (for details, see Table S2). The sections were observed and captured with the optical microscope (Leica DM 5000 B + CTR 5000) with UV lamp and saved with IM 1000 software. Densitometric analysis of the immunofluorescent signal was performed with ImageJ Software counting 25 seminiferous tubules/animal for a total of 150 tubules per group. Two different negative controls were performed: (1) by using rat isotype IgG (#I5006, Sigma-Aldrich; Milan, Italy) and (2) by omitting the primary antibody.

### 2.8. Statistical Analysis

Data were reported as mean ± standard error (SEM). Differences between the groups were considered statistically significant at *p* < 0.05. Analyses were performed using one-way ANOVA; Tukey’s post hoc *t*-test was applied when appropriate with Prism 5.0, GraphPad Software (San Diego, CA, USA).

## 3. Results

### 3.1. Histological Study

**Figure 1:**
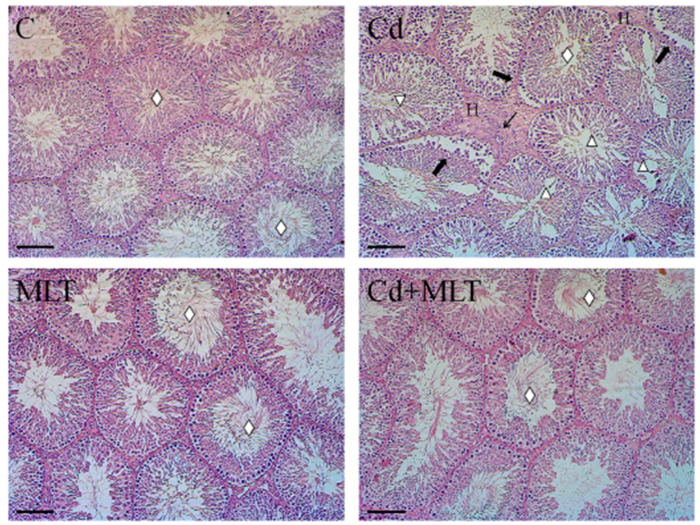
Hematoxylin-eosin staining of C-, Cd-, and/or MLT-treated rat testis. Evaluation of testicular histology of animals exposed to Cd and/or MLT. Rhombus: tubules lumen; Thick arrow: GC desquamation; Triangle: space between GC; Thin arrow: mononuclear cell infiltration; H: hemorrhage. Scale bars represent 40 µm.

Representative photomicrographs of testicular sections are shown in Figure 1. 

The control and MLT-treated rats presented a regular seminiferous epithelium and interstitial compartment characterized by the presence of germ cells (GC) in all the different stages of differentiation, with the tubular lumina being filled with mature SPZ (rhombus) and by the Leydig cells (LC) and normal blood vessels in the interstitium. Abnormal seminiferous tubules, with a general disorganization of the epithelium, were obvious in Cd-treated group, as indicated by the loss of contact and the presence of abundant empty spaces between the cells (triangle) other than desquamation of spermatogenic cells from the basement membrane (thick arrow), hemorrhage (H), and infiltrations of some mononuclear cells in the interstitial compartment (thin arrow). In the Cd+MLT-treated rats, the MLT preventive action was evidenced by a similar picture of the histological features, where the organization of the germinal and interstitial compartments was quite like that observed in the control testis.

The histological data were confirmed by the analysis of three morphometric parameters (Table 1), which showed that the tubules diameter (*p* < 0.001), the epithelium thickness (*p* < 0.001), and the percentage of tubular lumina occupied by SPZ (*p* < 0.001) were significantly lower in Cd-treated group as compared to the control and MLT groups. MLT prevented the Cd toxic effect on the epithelium thickness and percentage of empty lumina, which were comparable to those of the control; however, its action was less evident on the tubular diameter, which increased as compared to the Cd-treated group (*p* < 0.001), but not comparable to the control (*p* < 0.01). 

### 3.2. Oxidative Stress Markers

#### 3.2.1. CAT and SOD Activities

Cd treatment induced a significant reduction in testicular CAT activity when compared to that of the control (*p* < 0.001) and MLT (*p* < 0.001) groups (Table 2). 

No reduction was detected in Cd+MLT group, while, interestingly, MLT alone increased CAT activity as compared to the control level (*p* < 0.001). Similarly, testicular SOD activity significantly decreased (*p* < 0.001) in Cd-treated rats when compared to the control (*p* < 0.001) and MLT (*p* < 0.001) groups (Table 2). No difference between control, MLT, and Cd+MLT groups was observed. 

#### 3.2.2. Lipid Peroxidation

The Cd-induced oxidative stress was further assayed by analyzing the testicular lipid peroxidation level using the TBARS assay (Table 2). Cd exposure induced an increase of TBARS levels (*p* < 0.001) when compared to that of the control (*p* < 0.001) and MLT (*p* < 0.001) groups. MLT alone (*p* < 0.001) or given in combination with Cd (*p* < 0.01) attenuated the observed increase in the Cd group; however, the co-treatment did not completely restore the control value (*p* < 0.05). Unexpectedly, MLT induced a slight decrease in the TBARS levels compared to that of the control (*p* < 0.05).

### 3.3. Effect of Cd and/or MLT on Apoptosis

**Figure 2:**
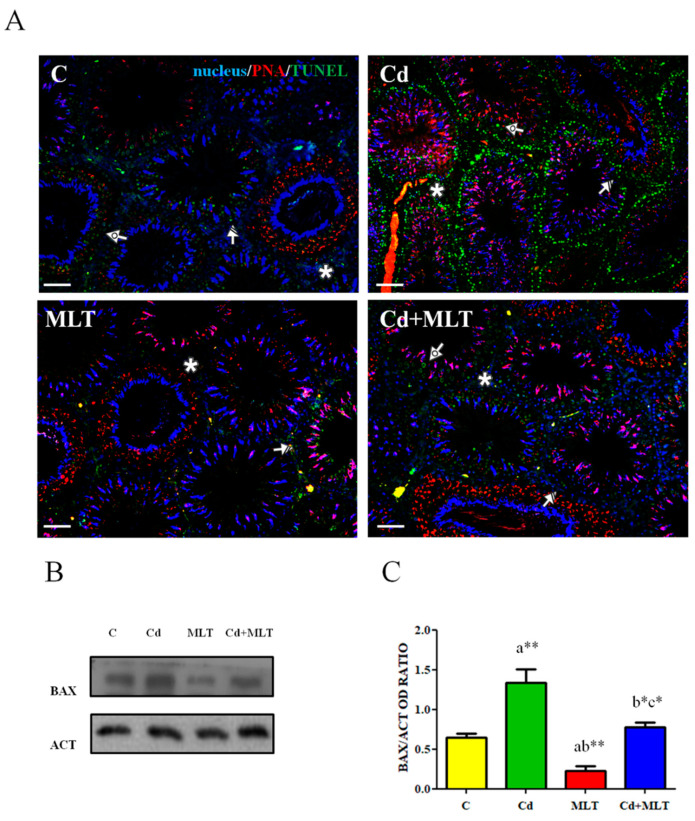
Apoptosis rate analysis of C-, Cd-, and/or MLT-treated rat testis. (**A**) Determination of apoptotic cells through the detection of TUNEL-positive cells (green) in the testes of animals treated with Cd and/or MLT. Slides were counterstained with DAPI-fluorescent nuclear staining (blue) and with PNA lectin (red), which marks the acrosome. Scale bars represent 20 μm. Striped Arrows: SPG; Dotted Arrows: SPC; Asterisks: LC. (**B**) WB analysis showing the expression of BAX (21 kDa) and β-actin (44 kDa) in the testes of animals treated with Cd and/or MLT. (**C**) Histograms showing the relative protein levels. Data were normalized with β-actin and reported as OD ratio using ImageJ. Values are expressed as means ± SEM from 6 animals in each group. a: significant difference versus C group, *p* < 0.05; b: significant difference versus Cd group, *p* < 0.05; c: significant difference versus MLT group, *p* < 0.05; *: *p* < 0.01; **: *p* < 0.001.

In Figure 2 is shown the effect of Cd and/or MLT administration on the apoptotic rate of germinal and somatic cells. 

Cd exposure produced a conspicuous increase in TUNEL-positive cells (Figure 2A), particularly spermatogonia (SPG) and spermatocytes (SPC; striped and dotted arrows, respectively), related to the control; scattered positive LC (asterisks) were also seen in the interstitial compartment. MLT alleviates the Cd-induced apoptosis since no differences were observed in the TUNEL-positive cells between control, MLT, and Cd+MLT groups.

To verify the involved apoptotic pathway, a WB analysis on BAX was performed (Figure 2B). Cd-exposure induced an increase of BAX protein level when compared to the control (*p* < 0.001) and MLT (*p* < 0.001) groups. No differences were found between control and Cd+MLT (*p* < 0.01) groups (Figure 2C), while the MLT antiapoptotic action was evidenced by the decrease of BAX protein level as compared to the control (*p* < 0.05).

### 3.4. Effect of Cd and/or MLT Testicular Steroidogenesis

**Figure 3:**
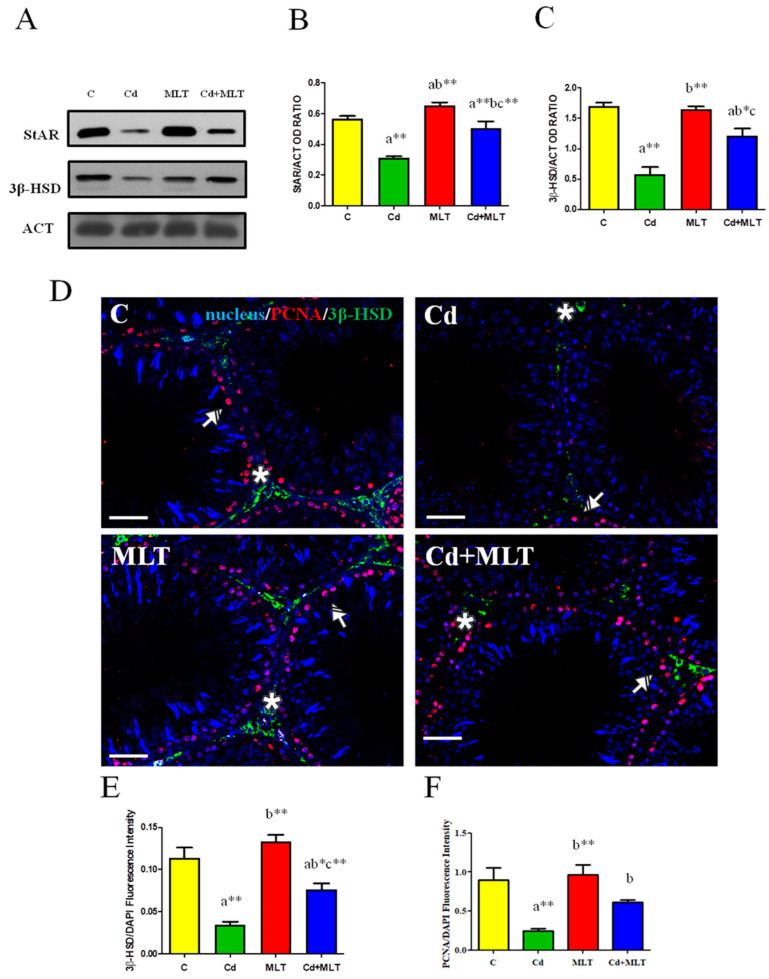
Steroidogenesis analysis of C-, Cd-, and/or MLT-treated rat testis. (**A**) WB analysis showing the expression of StAR (31 kDa), 3β-HSD (42 kDa), and β-actin (44 kDa) in testes of animals treated with Cd and/or MLT. (**B**,**C**) Histograms showing the relative protein levels of StAR and 3β-HSD, respectively. Data were normalized with β-actin and reported as OD ratio. (**D**) IF analysis of 3β-HSD (green) and PCNA (red) in testes of animals treated with Cd and/or MLT. Slides were counterstained with DAPI-fluorescent nuclear staining (blue). Scale bars represent 20 μm. Striped Arrows: SPG; Asterisk: LC. (**E**,**F**) Histogram showing the quantification of 3β-HSD and PCNA fluorescence signal intensity, respectively, using ImageJ. All the values are expressed as means ± SEM from 6 animals in each group. a: significant difference versus C group, *p* < 0.05; b: significant difference versus Cd group, *p* < 0.05; c: significant difference versus MLT group, *p* < 0.05; *: *p* < 0.01; **: *p* < 0.001.

For the evaluation of Cd toxicity on the testicular steroidogenesis, StAR and 3β-HSD protein levels and localization were assessed (Figure 3). 

Data indicated that Cd exposure significantly reduced the levels of both proteins as compared to that of control (*p* < 0.001). Furthermore, MLT ameliorated Cd effects, increasing StAR (*p* < 0.05; Figure 3A,B) and 3β-HSD levels (*p* < 0.01; Figure 3A,C) when compared to the Cd-treated animals, but the increase did not reach that of the control (*p* < 0.001 and *p* < 0.05, respectively; Figure 3B,C). Interestingly, MLT alone produced a slight increase of StAR protein levels as compared to that of the control (*p* < 0.05; Figure 3A,B). 

The alteration of steroidogenesis was further confirmed by 3β-HSD IF staining, shown in Figure 3D. For all the analyzed groups, the signal was specifically localized in the interstitial LC (asterisks, Figure 3D), but it was weak in the Cd-treated testis. Analysis of the fluorescence intensity showed a comparable pattern, statistically significant, as observed for the protein level (Figure 3E; legend), confirming the protective action of MLT on the Cd-induced impaired steroidogenesis.

Concurrently, labeling of PCNA, an S-phase cycle marker, was performed. Data showed its specific localization in the SPG layer at the basal tubules in all the groups (striped arrows; Figure 3D). Fluorescence intensity analysis indicated that Cd treatment modulated PCNA localization since a lower fluorescent signal was evidenced in the Cd group as compared to the control (*p* < 0.001) and MLT (*p* < 0.001) groups (Figure 3F). MLT ameliorated Cd effects, increasing PCNA fluorescence intensity when compared to the Cd-treated animals (*p* < 0.05) and was not different from those of the control. 

### 3.5. Effect of Cd and/or MLT on DAAM1 and PREP

#### 3.5.1. RT-PCR and WB Analysis

**Figure 4:**
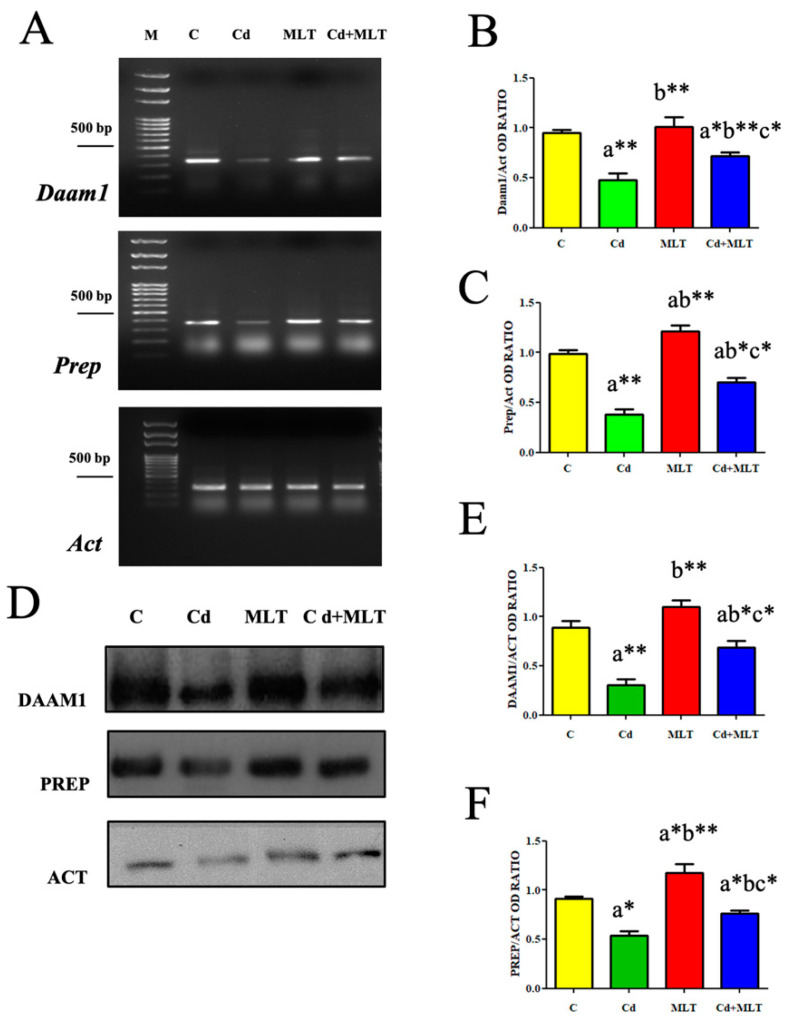
DAAM1 and PREP gene and protein expression of C-, Cd-, and/or MLT-treated rat testis. (**A**) Agarose gel electrophoresis of RT-PCR products showing the expression of *Daam1* (380 bp), *Prep* (392 bp), and *β-actin* (300 bp) in testes of animals treated with Cd and/or MLT. M: represents base-pairs marker (Solis BioDyne, Tartu, Estonia). (**B**,**C**) Histograms showing the relative mRNA levels of *Daam1* and *Prep*, respectively. Data were normalized with β-*actin* and reported as OD ratio. (**D**) WB analysis showing the protein levels of DAAM1 (112 kDa), PREP (80 kDa), and β-actin (44 kDa) in testes of animals treated with Cd and/or MLT. (**E**,**F**) Histograms showing the relative protein levels of DAAM1 and PREP, respectively. Data were normalized with β-actin and reported as OD ratio. All the values are expressed as means ± SEM from 6 animals in each group. a: significant difference versus C group, *p* < 0.05; b: significant difference versus Cd group, *p* < 0.05; c: significant difference versus MLT group, *p* < 0.05; *: *p* < 0.01; **: *p* < 0.001.

To verify Cd and/or MLT effects on the cytoskeleton of testicular cells, RT-PCR and WB analysis were performed on the formin DAAM1 and the peptidase PREP (Figure 4). 

Semi-quantitative RT-PCR is a specific technique, although it is less sensitive as compared with real-time PCR, that allows one to appreciate gene expression differences, just when these are evident and stable. Using RNA from the whole testis, we considered this method to robustly run correlation tests of gene expression (Figure 4A,C). Densiometric analysis of RT-PCR products showed that *Daam1*/*Act* and *Prep*/*Act* ratios decreased mRNA levels as compared to those of the control (*p* < 0.001; Figure 4B,C). Moreover, the co-administration of MLT increased *Daam1* and *Prep* levels when compared to the Cd-treated animals (*p* < 0.001 and *p* < 0.01, respectively) but not at the control level (*p* < 0.05). Interestingly, MLT alone produced a slight increase in *Prep* mRNA level as compared to that of the control (*p* < 0.05) 

WB analysis revealed that Cd exposure decreased DAAM1 protein level as compared to the control (*p* < 0.001) and MLT (*p* < 0.001) groups (Figure 4D,E). Furthermore, the co-administration of MLT increased DAAM1 levels when compared to the Cd-treated animals (*p* < 0.01), while no differences as compared to the controls were observed. Concerning PREP, Cd exposure decreased its protein levels as compared to the control (*p* < 0.01) and MLT (*p* < 0.001) groups (Figure 4D,F). The co-administration of MLT increased PREP levels when compared to the Cd-treated animals (*p* < 0.05), but the increase did not reach that of the control (*p* < 0.01). Interestingly, MLT alone also produced an increase of PREP protein levels compared to that of the control (*p* < 0.01).

#### 3.5.2. Immunofluorescence Analysis

**Figure 5:**
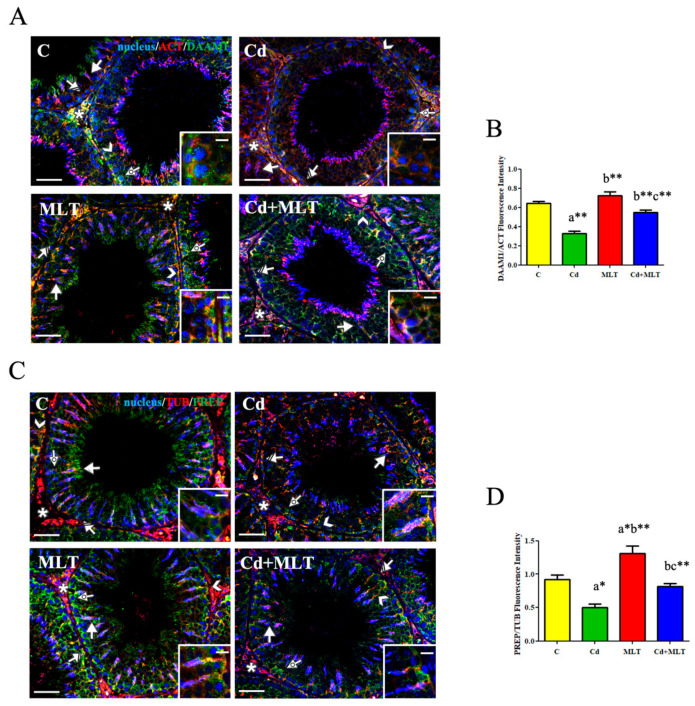
IF analysis on DAAM1 and PREP of C-, Cd-, and/or MLT-treated rat testis. (**A**) IF analysis of DAAM1 (green) and β-actin (red) in testes of animals treated with Cd and/or MLT. Slides were counterstained with DAPI-fluorescent nuclear staining (blue). Scale bars represent 20 μm. (**B**) Histogram showing the quantification of DAAM1 fluorescence signal intensity using ImageJ. (**C**) IF analysis of PREP (green) and α-tubulin (red) in testes of animals treated with Cd and/or MLT. Slides were counterstained with DAPI-fluorescent nuclear staining (blue). Scale bars represent 20 μm and 10 μm in the insets. Striped Arrows: SPG; Dotted Arrows: SPC; Arrows: SPT; Arrowheads: SC; Asterisk: LC. (**D**) Histogram showing the quantification of PREP fluorescence signal intensity using ImageJ. All the values are expressed as means ± SEM from 6 animals in each group. a: significant difference versus C group, *p* < 0.05; b: significant difference versus Cd group, *p* < 0.05; c: significant difference versus MLT group, *p* < 0.05; *: *p* < 0.01; **: *p* < 0.001.

To localize DAAM1 and PREP in the testes of Cd- and/or MLT-treated rats, a double IF staining together with their cytoskeletal partner (actin and tubulin, respectively), was performed (Figure 5). 

As shown in Figure 5A, in the control and MLT-treated testis, DAAM1 localized in the cytoplasm of SPG (striped arrows; insets), SPC (dotted arrows), and spermatids (SPT; arrows). Moreover, the results revealed a co-localization of DAAM1 with β-actin in Sertoli cells’ (SC) cytoplasm (arrowheads), highlighted by the intermediate yellow-orange tint. A positive staining was also found in the LC (asterisks). In the testis sections of Cd-exposed animals, a drastic decrease of staining in most germ cells (GC) was observed, while a weak signal was still present in the perinuclear zone of SPC (dotted arrows) and in SC cytoplasm (arrowheads). Interestingly, MLT counteracted the Cd effects on DAAM1 since no differences were found between control and Cd+MLT groups. The analysis of fluorescence intensity showed a comparable statistically significant pattern, as previously indicated for the protein level (Figure 5B; legend), confirming the protective action exerted by MLT on DAAM1 modulation provoked by Cd treatment.

As shown in Figure 5C, in the control and Cd+MLT-treated testis, PREP localized, to varying extent, in all cell types, and its immunopositivity was prevalently localized within the SPT (arrows) and SC cytoplasmic protrusions, where the co-localization with tubulin, highlighted by the intermediate yellow-orange tint, was evident (arrowheads; insets). In the testicular sections of Cd-exposed animals, PREP-localization pattern was comparable to that of the control and, although a strong decrease of staining in most GC was observed, the signal was still evident in the SC cytoplasm (arrowheads). Interestingly, in the MLT-treated group, PREP localized in the same above-mentioned cells, showing a fluorescent signal noticeably more intense compared to that of the control. Indeed, the fluorescence intensity analysis revealed a similar pattern, statistically significant, as observed for the protein level, confirming not only the protective action of MLT on PREP but also that MLT alone increased its protein levels (Figure 5D; legend).

## 4. Discussion

Mammalian spermatogenesis is a developmental process that involves a proliferative (both mitotic and meiotic) and a differentiative phase of GC, comprising a sequence of biochemical and morphological modifications to generate functional SPZ. Each step is harmonized not only by a cell type- and stage-specific regulation of the expression of precise genes, but it is also coordinated by an intricate network of endocrine, paracrine, and autocrine factors to produce good-quality gametes as an essential feature for reproductive success [37,38]. Nowadays, a severe decline in SPZ quality and quantity is occurring, above all, in the most industrialized countries, suggesting that the environmental release of many pollutants is one of the principal causes contributing to a reduction of fertility [2,3]. Cd is one of the most threatening environmental and occupational chemicals due to its prolonged persistence (approximately 30 years) and, consequently, its accumulation in several tissues [39], including male and female reproductive systems [40]. Since exposure to Cd is unavoidable beyond to expand upon the knowledge concerning its induced reprotoxicity, it is significant to explore strategies that may prevent, alleviate, or counteract Cd effects. In this regard, we previously demonstrated that MLT is capable of counteracting Cd-induced toxicity in rat bone [21] and ovary [22], especially because of its antioxidant properties [28]. To our knowledge, very few papers explored the beneficial role of MLT against testicular Cd toxicity [41,42,43,44,45]; here, we further confirmed the antioxidant and antiapoptotic action of MLT, also documenting the ameliorative effect of MLT on other parameters that have never been analyzed before, i.e., the protein level of steroidogenic enzymes and the expression of the cytoskeleton-associated proteins DAAM1 and PREP. Noteworthy, adverse influence of Cd on testicular function is probably due to induced oxidative stress, supported by the increased testicular TBARS levels, an index of the lipid peroxidation [33], and by the decreased activity of the antioxidant enzymes CAT and SOD. Per se, Cd is unable to generate free radicals; indeed, it has been proposed that the induced oxidative stress may occur through two mechanisms: (1) via the so-called phenomenon of the molecular mimicry, whereby Cd replaces other metals, such as iron and copper, from cytoplasmic and membrane proteins, thus increasing their concentrations; this, in turn, induces ROS overproduction by the Fenton reaction [46]; and (2) Cd interferes with the –SH groups within the active sites of the antioxidant enzymes, inducing the misfolding, and, finally, affecting their activity [47]. Considering that testicular cells have a high content of unsaturated fatty acids [48], testes are extremely susceptible to ROS [49]. Although a moderate amount of ROS is opportune for GC physiology [50], their overproduction induces histological alterations and apoptosis in the seminiferous epithelium [51,52]. This last point was further confirmed by the increased level of BAX, a pro-apoptotic protein which, by inducing the opening of the mitochondrial voltage-dependent anion channel (VDAC), stimulates the intrinsic apoptotic cascade [53]. These findings confirm a previous report in which Cd impairment of spermatogenesis occurs via enhanced apoptosis, interfering with VDAC and activating JNK/p53 signaling [54]. Ultimately, the increased number of TUNEL-positive GC, together with the lower amount of PCNA-stained cells, may be the cause of the reduced SPZ concentration observed in Cd-treated animals.

Interestingly, scattered apoptotic LC were also present, confirming the endocrine-disrupting action exerted by Cd [55]. Indeed, it accumulates in neuroendocrine tissues and interferes with the HPT axis [56,57]; thus, the altered protein levels of StAR and 3β-HSD, key enzymes involved in testosterone (T) biosynthesis, may consequently have an impaired steroid availability and production at the central and local level, resulting in the reduction in serum and testicular T concentrations [7]. It must be pointed out that T regulates the whole spermatogenetic process, acting as a “survival factor” to protect GC from apoptosis, and its depletion may be an additional cause of the augmented apoptosis, together with the alteration of the histological structure [58]. Data confirmed that MLT effectively reduced oxidative stress and Cd-induced apoptosis, underlined by the reduced TBARS concentration and the increased CAT and SOD antioxidant activity as well as the reduced number of TUNEL-positive cells and BAX protein levels, respectively.

Many reports in the field of the reproductive toxicology demonstrated that one of the main targets of environmental pollutants, and particularly Cd, are the events controlling the dynamics of the cytoskeleton of both somatic and germ cells.

Furthermore, findings coming from the use of toxicant models are helpful tools to explore not only the toxic effects of these substances on the normal spermatogenesis but also to understand the specific cellular events associated with the proliferation and differentiation of testicular cells since the regulation of microfilaments and microtubules dynamics during the epithelial cycle is still poorly understood [59].

Thus, for a better understanding of the picture of Cd effects, the analysis was extended to two proteins that we previously demonstrated to be involved in the cytoskeletal dynamics associated with the male germ compartment and reproduction: DAAM1 [15,60] and PREP [60,61]. DAAM1 belongs to the formin family and regulates the nucleation of unbranched actin filaments [36]; PREP is a serine protease, and it has also been associated with microtubules [62]. Cd treatment, thanks to its ability to influence the activity of many transcription factors and transduction signals [46], provokes changes in their expression. Since DAAM1 and PREP are involved in the intimate changes in GC morphology during their differentiation into mature SPZ, regulating the homeostasis of actin and microtubules organization, we hypothesize that the observed altered histological parameters, such as desquamation of GC, may be due to an impaired expression of these proteins induced by the Cd treatment, leading to alteration of cytoskeleton dynamics. Interestingly, in our previous work, we found an increase of PREP expression [20]; conversely, here, an opposite effect of Cd treatment on PREP was documented. This could be explained with differences in the methodological approach and also in the age of the rats. In fact, it has been proven that there is an interaction between Cd exposure and its age-dependent effects on the HPT axis function [56,57,63]. Cd exposure, in different ways, disturbs reproductive hypothalamic and pituitary hormonal levels, depending on the age of the animals, in relation to its selective accumulation within the HPT axis [56,57,63]. Thus, Cd may exert varying toxic effects on mature or developing mature gonads and HTP axis [20]. In addition, although scarce information on the testis is present in the literature, many papers reported that, during embryonal and post-natal development, PREP expression and activity changes in the rat brain [64,65,66]. Finally, considering that PREP levels are physiologically different during pre- and post-natal life, the above-mentioned contrasting results may be attributed just to the described diverse Cd action during rats’ development together with the variations of PREP levels.

Here, again, MLT co-treatment ameliorated Cd effects on DAAM1 and PREP expression, even if it was not able to completely restore to the control values. This could be by a direct effect of MLT on cytoskeleton modulation [67] or indirectly by decreasing the oxidative stress, which is Cd-induced, that notoriously influences the normal cytoskeleton physiology [68,69,70,71].

Another interesting result came from the treatment using MLT alone, in fact, the observation that it was able to induce an increased PREP expression. PREP, beyond its involvement in microtubule-associated processes, participates in many other cellular phenomena thanks to its peptidase activity [72]. One of these is the control of the hormonal homeostasis in regulating TRH, GnRH, and progesterone concentrations. Of note, data by Xu et al. [73] demonstrating that PREP increased the expression of the steroidogenic enzymes, StAR, and 3β-HSD via the ERK signaling pathway in murine luteal cells well support our results since we also observed the increase of StAR protein level induced by MLT treatment. Thus, we may hypothesize that MLT regulates testosterone synthesis just acting on PREP expression; however, further studies are still required to confirm this point.

## 5. Conclusions

Here is confirmed the ameliorative role of MLT on Cd-induced reprotoxicity in the rat testis by reducing oxidative stress and the augmented apoptosis. However, we added new insights into the mechanism related to the protective action of MLT in all the other considered testicular parameters, i.e., the impaired steroidogenesis, as highlighted by the reduced protein level of StAR and 3β-HSD. Moreover, for the first time, we showed a MLT counteractive effect also on the cytoarchitecture of somatic and germ cells since Cd perturbed the normal cytoskeletal dynamics reducing DAAM1 and PREP expression. Finally, MLT alone can induce PREP expression and, consequently, augment protein level of StAR, regulating sex steroids production.

The combined results strongly denote a role of MLT in improving rat testicular health; however, further studies are needed to verify its action in humans affected by fertility disorders to produce high-quality SPZ to favor reproduction.

## Figures and Tables

**Table 1:** Effect of Cd and/or MLT on testicular morphometric parameters.

Groups	C	Cd	MLT	Cd-MLT
Tubules Diameter (µm)	168.19 ± 29.81	126.69 ± 21.52 ^a**^	162.22 ± 38.26 ^b**^	145.67 ± 19.38 ^a*b**c**^
Epithelium Thickness (µm)	46.01 ± 16.31	31.66 ± 9.74 ^a**^	48.29 ± 12.22 ^b**^	44.58 ± 13.95 ^b**^
Empty Lumen (%)	56.1 ± 1.75	77 ± 2.2 ^a**^	57.9 ± 3.6 ^b**^	61.8 ± 4.9 ^b^

Evaluation of testicular morphometric parameters of animals exposed to Cd and/or MLT. Values are expressed as mean ± SEM from 6 animals in each group. a: significant difference versus C group, *p* < 0.05; b: significant difference versus Cd group, *p* < 0.05; c: significant difference versus MLT group, *p* < 0.05; *: *p* < 0.01; **: *p* < 0.001.

**Table 2:** Effect Cd and/or MLT on testicular oxidative-stress parameters.

Groups	C	Cd	MLT	Cd-MLT
CAT Activity (U/mg of proteins)	37.52 ± 1.07	24.46 ± 7.25 ^a**^	49.64 ± 0.88 ^a**b**^	33.77 ± 1.18 ^b**c**^
SOD Activity (U/mg of proteins)	15.17 ± 0.07	8.86 ± 0.13 ^a**^	16.38 ± 0.08 ^b**^	12.58 ± 0.05 ^bc^
[TBARS] (µM/µg of proteins)	0.1092 ± 0.002	0.1578 ± 0.006 ^a**^	0.0894 ± 0.004 ^ab**^	0.1306 ± 0.003 ^ab*c**^

CAT and SOD enzymatic activities and TBARS levels of animals exposed to Cd and/or MLT. Values are expressed as mean ± SEM from 6 animals in each group. a: significant difference versus C group, *p* < 0.05; b: significant difference versus Cd group, *p* < 0.05; c: significant difference versus MLT group, *p* < 0.05; *: *p* < 0.01; **: *p* < 0.001.

## Data Availability

The data presented in this study are available in the article and its Supplementary Materials.

## Supplementary Materials

The following are available online at https://www.mdpi.com/article/10.3390/genes12071016/s1. Table S1: List of all the used primers antibodies.
