# Supplementary material for: Altered Expression of DAAM1 and PREP Induced by Cadmium Toxicity Is Counteracted by Melatonin in the Rat Testis
Source: Genes (Basel). 2021 Jun 30;12(7):1016. doi: 10.3390/genes12071016 (PMC8304460; doi:10.3390/genes12071016)
Supplement: Supplementary file 1 [file genes-12-01016-s001.zip › genes-1267409-supplementary.pdf]

Table S1. List of all the used primers and antibodies

Gene	GenBank Accession Nr.	Annealing Temperature	Amplicon size (bp)	Primers Sequence
Daam1	NM_001108030.1	56° C	380	For: 5'- CCGAAACAATGACCACCCAG -3'<br>Rev: 5'- GCTTTGTCCTCAGTGCTGTC -3'
Prep	NM_031324.2	55° C	392	For: 5'- CCCTTATGCTTGGCTTGAAG -3'<br>Rev: 5'- TCATGAACTTGATGGTCACC -3'
β-Actin	NM_031144.3	56° C	300	For: 5'- CTCTTCCAGCCTTCCTTCCT -3'<br>Rev: 5'- CTGCTTGCTGATC-CACATC -3'

Antibody	WB Dilution	IF Dilution	Source
3β-HSD	1:700	1:100	Elabscience Biotechnology, Wuhan, China #E-AB-15112
α-Tubulin	-	1:100	Elabscience Biotechnology, Wuhan, China #E-AB-20036
β-Actin	1:5000	1:100	Elabscience Biotechnology, Wuhan, China #E-AB-20031
BAX	1:250	-	Santa-Cruz Biotechnology, Inc., Dallas, TX, USA (#sc-526)
DAAM1	1:500	1:100	Elabscience Biotechnology, Wuhan, China #E-AB-13182
PCNA	-	1:100	Santa-Cruz Biotechnology, Inc., Dallas, TX, USA #sc-56
PREP	1:3000	1:100	Abcam, Cambridge, UK #ab58988
StAR	1:500	-	Elabscience Biotechnology, Wuhan, China #E-AB-15419
PNA lectin Alexa Fluor 568	-	1:50	Thermo Fisher Scientific, Waltham, Ma, USA #L32458
Goat anti-rabbit HRP	1:5000	-	Sigma-Aldrich, Milan, Italy #AP307P
Goat anti-mouse HRP	1:5000	-	Sigma-Aldrich, Milan, Italy #AP130P
Goat anti-rabbit Alexa Fluor 488	-	1:500	Thermo Fisher Scientific, Waltham, Ma, USA #A32731
Goat anti-mouse Alexa Fluor 647	-	1:500	Thermo Fisher Scientific, Waltham, Ma, USA #A21236
